# Blockchain-enabled traceability evaluation framework for mineral resource development and utilization: a fuzzy comprehensive assessment approach

**DOI:** 10.1038/s41598-026-40195-1

**Published:** 2026-03-02

**Authors:** Guodong Ma, Hongxi Bai, Wei Zhao, Qicheng Yun, Yu Zhang

**Affiliations:** 1 Mineral Resources Evaluation Department, Qinghai Geological Survey, Xining, 810000 Qinghai China; 2 Technical Department, Qinghai Geological Survey Bureau, Xining, 810000 Qinghai China

**Keywords:** Blockchain technology, Mineral resource traceability, Evaluation framework, Fuzzy comprehensive evaluation, Smart contracts, Supply chain management, Engineering, Mathematics and computing

## Abstract

Effective traceability management in mineral resource development faces persistent challenges including information asymmetry, data falsification, and verification difficulties across complex value chains. This paper proposes a comprehensive blockchain-enabled traceability evaluation framework integrating distributed ledger technology with systematic assessment methodologies. A four-layer architecture encompassing data acquisition, blockchain storage, analysis processing, and evaluation application is designed to ensure data integrity throughout the mineral lifecycle. A hierarchical indicator system spanning five dimensions—traceability breadth, depth, precision, timeliness, and data credibility—is constructed, with the Analytic Hierarchy Process employed for weight determination and fuzzy comprehensive evaluation applied for performance assessment. Empirical validation through case study analysis of Huaxin Mining Group demonstrates the framework’s practical applicability, yielding a comprehensive traceability score of 81.2 (Good grade). Comparative analysis reveals that blockchain-based systems achieve 96.8% data accuracy versus 82.4% for traditional approaches, with trace-back efficiency improving from 127.3 min to 4.7 min. The blockchain technology contribution ratio reaches 47.3% toward maximum traceability improvement. These findings provide theoretical foundations and practical guidance for advancing transparent and accountable mineral resource governance.

## Introduction

Mineral resources constitute the bedrock of industrial progress and economic vitality worldwide. Yet the development and utilization of these resources confront escalating difficulties that imperil sustainable governance practices. A careful examination of contemporary mineral resource management reveals several persistent governance challenges:

- Information asymmetry pervades relationships among mining enterprises, regulatory bodies, and end consumers, eroding oversight effectiveness and market trust^1^.
- Data falsification occurs throughout extraction, processing, and transportation phases, where actors sometimes manipulate production figures, environmental compliance documentation, and quality certifications to evade regulatory scrutiny^2^.
- Verification difficulties arise from fragmented documentation systems and jurisdictional gaps, complicating efforts to authenticate material provenance across complex value chains.
- Cross-border regulatory coordination remains weak, as inconsistent standards and limited data sharing between jurisdictions create exploitable loopholes.
- Traditional monitoring approaches, which depend heavily on manual inspections and paper-based records, cannot match increasingly sophisticated circumvention strategies^3^.

These governance deficits extend well beyond administrative inconvenience. They strike at the heart of resource governance integrity, permitting illicit materials to infiltrate legitimate supply streams while undermining consumer confidence and regulatory authority.

### Research background and literature review

The emergence of blockchain technology has sparked considerable interest among researchers seeking solutions to these persistent governance challenges. Scholars have explored blockchain applications across supply chain management, demonstrating its potential for establishing immutable transaction records and enhancing data transparency^4^. Several investigations have extended these concepts to natural resource sectors, proposing distributed ledger architectures for tracking timber, fisheries, and agricultural commodities from origin to end-user^5^. Within the mining domain specifically, preliminary efforts have examined how blockchain might facilitate conflict mineral certification and responsible sourcing verification^6^.

Parallel research streams have focused on traceability management frameworks for resource exploitation. These studies emphasize the importance of capturing comprehensive lifecycle data spanning exploration, extraction, beneficiation, and distribution phases^7^. Evaluation methodologies for assessing traceability system performance have likewise attracted scholarly attention, with researchers proposing various indicator systems grounded in reliability, completeness, and accessibility criteria^8^. Some investigators have attempted to integrate environmental impact assessment protocols with traceability mechanisms, recognizing that sustainable resource governance requires holistic approaches^9^.

### Research gaps and motivation

Despite these advances, critical deficiencies persist in existing scholarship. Current frameworks frequently overlook the fundamental question of data credibility—the extent to which recorded information accurately reflects physical reality rather than fabricated inputs^10^. Many proposed systems assume trustworthy data sources without adequately addressing the technical and institutional mechanisms necessary to guarantee such trustworthiness. Furthermore, traceability completeness remains problematic, as information gaps often emerge during transitions between custody holders or jurisdictional boundaries. The scientific rigor of existing evaluation methodologies also warrants scrutiny; numerous studies rely on subjective expert judgments without establishing robust validation procedures^11^.

These limitations underscore the necessity of developing blockchain-enabled traceability evaluation frameworks specifically tailored to mineral resource contexts. Blockchain’s inherent characteristics—decentralization, immutability, transparency, and cryptographic security—offer promising pathways for addressing data integrity concerns that have long plagued resource governance systems. The integration of smart contract functionality enables automated verification protocols that reduce reliance on potentially corruptible human intermediaries. From a theoretical perspective, such integration advances our understanding of how distributed trust architectures can transform natural resource management paradigms.

### Research objectives and innovations

This paper develops a comprehensive traceability evaluation framework for mineral resource development and utilization grounded in blockchain technology. Our investigation unfolds through three interconnected phases: theoretical framework construction, evaluation indicator design, and empirical verification via case analysis^12^. We distinguish our contributions along two dimensions, namely application novelty and methodological novelty.

Regarding application novelty, this study represents one of the early attempts to deploy a blockchain-based traceability evaluation system within a real-world mineral extraction context. The technology fusion approach integrates blockchain infrastructure with Internet of Things sensing devices and geographic information systems, enabling multi-source data acquisition and cross-validation in an operational mining environment.

Concerning methodological novelty, we construct a hierarchical indicator system spanning data authenticity, process completeness, stakeholder accessibility, and governance effectiveness. We further introduce hybrid evaluation methods that combine objective measurement with structured expert assessment, balancing scientific rigor and practical applicability.

It should be emphasized that our empirical validation rests on a single enterprise case study, and the contributions we claim are therefore best understood as case-based empirical evidence rather than generalizable theoretical foundations. The framework illustrates potential applicability to mineral resource governance and offers actionable guidance for practitioners, though multi-case validation remains necessary to establish broader generalizability.

## Theoretical foundation and technical architecture

### Blockchain technology principles and characteristic analysis

Blockchain technology originated with Nakamoto’s seminal 2008 white paper proposing a peer-to-peer electronic cash system^13^. What began as the underlying infrastructure for cryptocurrency has since evolved through distinct generational phases. The first generation focused primarily on digital currency applications, while subsequent iterations introduced programmable transaction logic and enterprise-grade scalability enhancements^14^. This technological maturation has expanded blockchain’s applicability far beyond financial services into domains requiring robust data integrity assurance.

At its core, blockchain operates as a distributed ledger system wherein transaction records are grouped into cryptographically linked blocks. Each block contains a hash pointer referencing its predecessor, creating an append-only chain structure resistant to retroactive modification. The hash function, typically SHA-256, transforms input data of arbitrary length into fixed-length output according to the following standard cryptographic formulation:

1 $$H\left( x \right)=SHA256\left( x \right) \to {\{ 0,1\} ^{256}}$$

Network participants achieve agreement on ledger state through consensus mechanisms—algorithmic protocols that coordinate distributed decision-making without centralized authority^15^. Proof-of-Work, Proof-of-Stake, and Practical Byzantine Fault Tolerance represent prominent consensus variants, each presenting distinct trade-offs between security guarantees, throughput capacity, and energy consumption.

Smart contracts extend blockchain functionality by enabling self-executing programmatic logic. These digital agreements automatically enforce predefined conditions when triggered by qualifying events. The execution outcome *O* of a smart contract can be expressed as a deterministic function:

2 $$O=f\left( {{S_t},{T_{input}}} \right) \to {S_{t+1}}$$

where ${S_t}$ denotes current state and ${T_{input}}$ represents the triggering transaction input.

The convergence of these technical components yields distinctive characteristics with profound implications for traceability applications. Decentralization eliminates single points of failure and reduces vulnerability to manipulation by individual actors^16^. Cryptographic chaining renders historical records practically immutable, as altering any block would invalidate all subsequent hash linkages. Transparency enables authorized stakeholders to independently verify recorded information. These properties have attracted substantial attention in supply chain contexts, where researchers have demonstrated blockchain’s capacity for enhancing product provenance verification and combating counterfeiting^17^. Resource extraction sectors have begun exploring similar applications, though implementation remains nascent compared to consumer goods industries^18^.

### Connotation of mineral resource development and utilization traceability

Traceability in mineral resource contexts refers to the systematic capacity to reconstruct the complete history, application pathways, and custody transitions of extracted materials across their lifecycle^19^. However, “traceability” itself encompasses distinct problem types that warrant clarification. Provenance reconstruction concerns the ability to trace materials backward to their geographic and temporal origins. Auditability under adversarial conditions addresses whether recorded data can withstand deliberate manipulation attempts and remain verifiable even when some actors behave dishonestly. Operational efficiency pertains to the speed and cost-effectiveness with which tracing queries can be executed. IT system performance relates to technical metrics such as throughput, latency, and uptime that characterize the underlying information infrastructure.

This paper focuses primarily on provenance reconstruction and data integrity assurance, which together enable stakeholders to identify accountability, verify compliance, and detect irregularities within complex value chains. We do not claim to fully address auditability under all adversarial scenarios, particularly those involving collusion among multiple parties or sophisticated physical tampering at data collection points. IT system performance metrics appear in our evaluation framework but serve as supporting indicators rather than the central objects of assessment. Readers should interpret our findings with these scope boundaries in mind.

The mineral resource lifecycle comprises interconnected phases, each generating distinct traceability elements. Exploration activities produce geological survey data, reserve estimations, and licensing documentation. Extraction operations yield production volumes, environmental monitoring readings, and safety compliance records. Processing stages transform raw ores into refined products, necessitating documentation of input-output ratios, quality parameters, and waste generation metrics^20^. Transportation introduces custody transfer records, logistics timestamps, and handling condition logs. Sales transactions complete the chain with commercial documentation linking physical commodities to financial flows.

Effective traceability management demands adherence to three core requirements. Information completeness ensures no lifecycle gaps exist—missing data points compromise investigative capacity. We can express the completeness index *C* as follows:

3 $$C=\frac{{\mathop \sum \nolimits_{{i=1}}^{n} {D_i}}}{{\mathop \sum \nolimits_{{i=1}}^{n} D_{i}^{{required}}}} \times 100\%$$

where ${D_i}$ represents documented data points and $D_{i}^{{required}}$ denotes mandatory documentation items^21^. Data authenticity guarantees that recorded information faithfully represents physical reality rather than fabricated inputs. Chain continuity maintains unbroken linkages between successive custody holders, preventing untraceable material from entering legitimate streams^22^.

Traditional traceability approaches rely predominantly on paper-based documentation and centralized databases. These methods suffer from inherent vulnerabilities: documents can be forged, database entries modified without detection, and information silos impede cross-organizational verification^23^. The verification confidence level *V* under traditional systems diminishes exponentially with chain length:

4 $$V={V_0} \times {e^{ - \lambda n}}$$

where ${V_0}$ represents initial confidence, $\lambda$ denotes decay coefficient, and *n* indicates the number of custody transfers. Blockchain technology addresses these deficiencies through cryptographic integrity assurance and distributed consensus validation, rendering it particularly suited for mineral traceability applications where trust deficits remain pervasive^24^.

### Theoretical framework for traceability evaluation

The construction of a rigorous traceability evaluation framework necessitates grounding in established theoretical foundations. Systems theory provides the overarching conceptual lens, treating mineral resource chains as complex adaptive systems wherein components interact dynamically across organizational boundaries^25^. This perspective emphasizes holistic assessment rather than fragmented examination of isolated elements. Information theory contributes analytical tools for quantifying data transmission fidelity and entropy reduction through systematic documentation. The entropy measure *H* for traceability information can be expressed using Shannon’s standard formula:

5 $$H\left( X \right)= - \mathop \sum \limits_{{i=1}}^{n} p\left( {{x_i}} \right){\mathrm{lo}}{{\mathrm{g}}_2}p\left( {{x_i}} \right)$$

where $p\left( {{x_i}} \right)$ represents the probability of information state ${x_i}$^26^. Lifecycle theory completes the theoretical triad by mandating cradle-to-grave perspectives that capture cumulative impacts across all operational phases.

Traceability evaluation demands multi-dimensional analysis recognizing that system performance manifests through diverse yet interrelated aspects. Table 1 summarizes the dimensional classification framework adopted in this study, delineating core evaluation perspectives alongside their constituent focus areas and representative indicators.

**Table 1:** Dimensional classification of traceability evaluation.

Dimension	Focus area	Core elements	Representative indicators
Technical	Infrastructure capability	Data architecture, consensus protocols	System throughput, latency metrics
Management	Governance effectiveness	Process standardization, stakeholder coordination	Compliance rates, audit frequency
Benefit	Value realization	Cost reduction, risk mitigation	Return on investment, incident reduction
Information	Data quality assurance	Accuracy, timeliness, accessibility	Error rates, update intervals

Multi-criteria decision theory offers methodological scaffolding for synthesizing evaluations across these heterogeneous dimensions^27^. The weighted aggregation approach permits integration of disparate performance measures into composite indices. Following the standard additive weighting formulation, the overall traceability performance score *S* is calculated as:

6 $$S=\mathop \sum \limits_{{j=1}}^{m} {w_j} \times {v_j}$$

where ${w_j}$ denotes dimension weight and ${v_j}$ represents normalized performance value for dimension *j*^28^. Weight determination itself presents methodological challenges, as subjective importance assessments may introduce evaluator bias. Analytic Hierarchy Process and entropy weighting methods provide complementary approaches for addressing this concern.

The blockchain-enabled traceability evaluation framework proposed herein integrates these theoretical strands into a coherent analytical structure. The framework operates through three sequential phases: indicator selection guided by lifecycle comprehensiveness criteria, data collection via blockchain-verified sources, and multi-criteria aggregation yielding performance scores^29^. The evaluation logic follows a bottom-up trajectory—individual indicators aggregate into dimensional scores, which subsequently combine into the overall traceability index *T*:

7 $$T=f\left( {{S_{tech}},{S_{mgmt}},{S_{benefit}},{S_{info}}} \right)$$

This formulation captures interdependencies while maintaining dimensional distinctiveness essential for diagnostic purposes^30^. The methodological foundation thus established enables systematic, reproducible, and theoretically grounded assessment of blockchain-based traceability systems in mineral resource contexts.

**Fig. 1:**
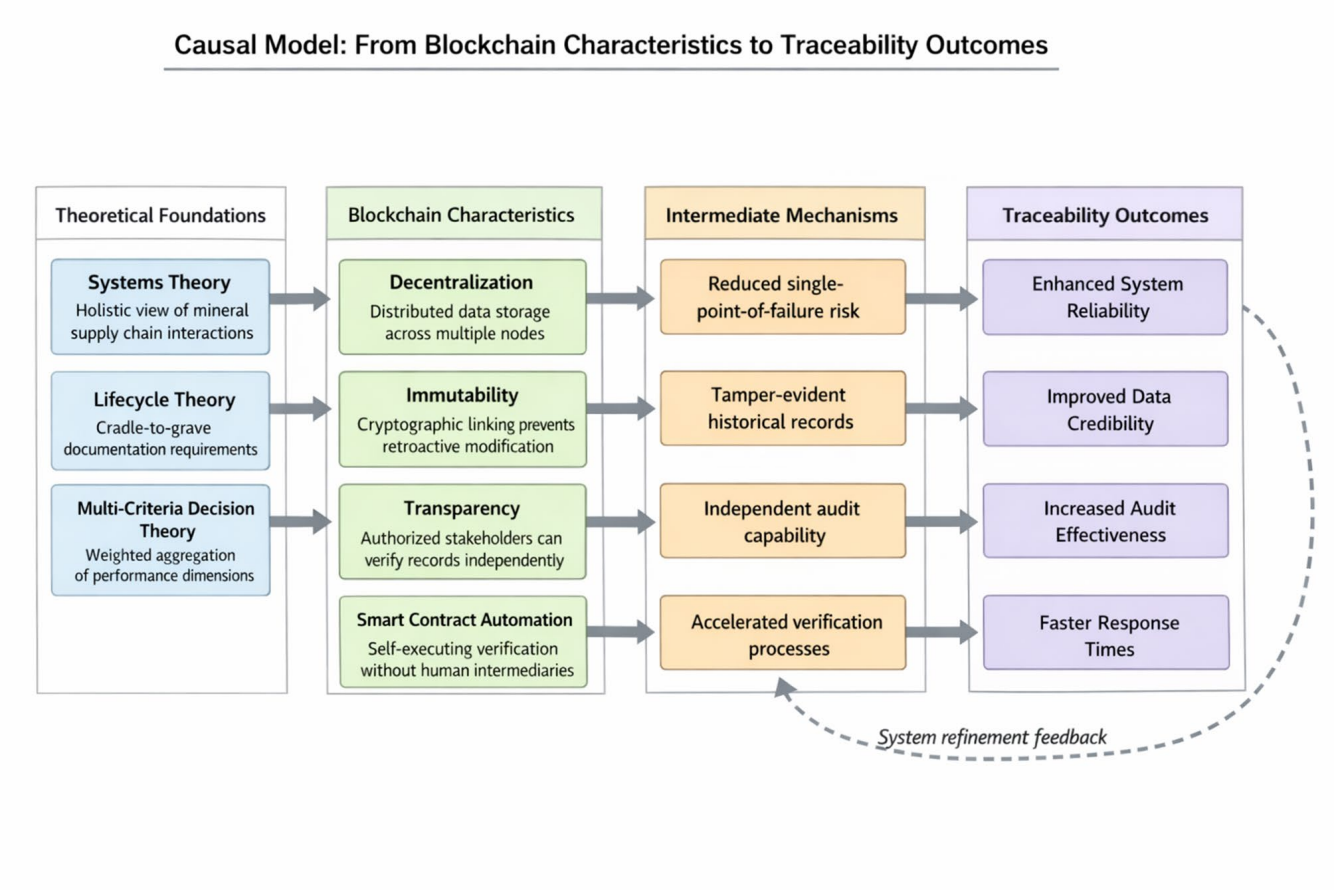
Causal model linking blockchain characteristics to traceability outcomes.

To clarify the logical relationships underpinning our evaluation approach, Fig. 1 presents a causal model linking blockchain characteristics to measurable traceability outcomes. The model illustrates how theoretical foundations (systems theory, lifecycle theory, and multi-criteria decision theory) inform framework design, which in turn determines blockchain feature deployment and ultimately produces observable traceability performance indicators.

The causal logic proceeds as follows. Decentralization distributes data storage and validation across multiple nodes, reducing vulnerability to localized failures or manipulation. Immutability ensures that once data enters the blockchain, modification becomes computationally infeasible without network consensus, thereby strengthening data credibility assessments. Transparency permits authorized stakeholders to independently verify recorded information, supporting auditability objectives. Smart contract automation executes predefined verification logic without manual intervention, accelerating response times and reducing opportunities for corrupt intermediation. These theoretical linkages guide our indicator selection and weight assignment in subsequent evaluation procedures.

## Construction of blockchain-based traceability evaluation framework

### Overall architecture design of evaluation framework

**Fig. 2:**
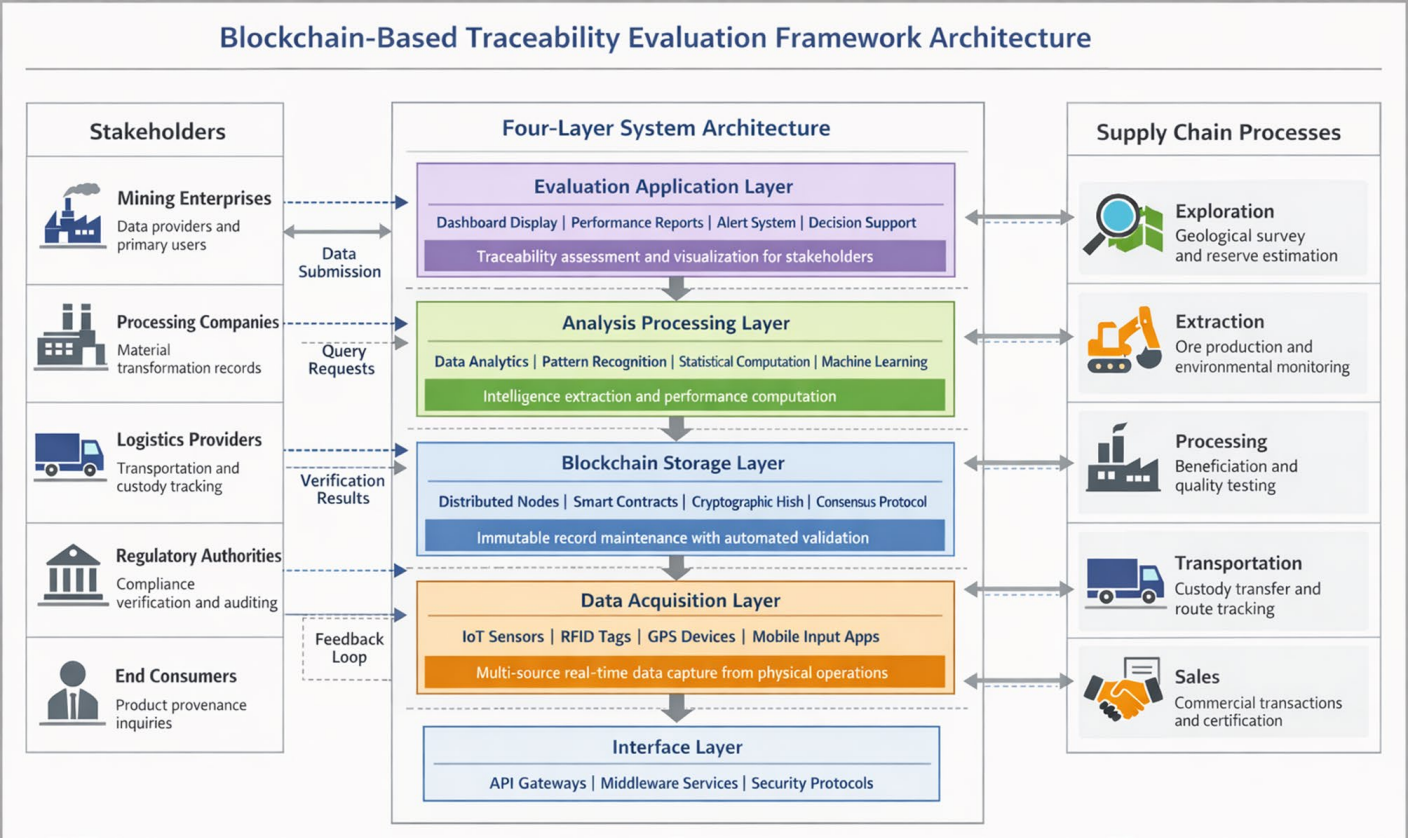
Overall architecture of blockchain-based traceability evaluation framework with stakeholder interactions.

The proposed evaluation framework adopts a layered architectural paradigm that separates functional concerns while maintaining seamless data integration across system components. This design philosophy reflects practical considerations—modular construction facilitates incremental deployment and targeted upgrades without disrupting operational continuity^31^. The architecture comprises four hierarchically organized layers, each addressing distinct aspects of the traceability evaluation process. As illustrated in Fig. 2, these layers interact through well-defined interfaces that govern data transformation and transmission protocols.

The architecture design draws inspiration from recent blockchain-based traceability implementations in related resource sectors. Chadly et al.^51^demonstrated a comprehensive system architecture for tracking rare earth metals in photovoltaic supply chains, incorporating stakeholder roles and smart contract interactions. Patro et al.^52^presented a carbon footprint traceability framework for aviation that illustrates effective integration of multiple data sources with blockchain verification mechanisms. Moawad et al.^53^developed an NFT-based tracking system for lithium-ion battery materials that addresses sustainability certification throughout the supply chain lifecycle.

Our architecture incorporates several design trade-offs worthy of explicit acknowledgment. First, we adopt a consortium blockchain model rather than a public chain, sacrificing some degree of decentralization in exchange for improved throughput and governance control appropriate to enterprise contexts. Second, the data acquisition layer employs a hybrid sensing strategy combining automated IoT devices with manual input interfaces, recognizing that full automation remains economically prohibitive in certain operational environments. Third, privacy-preserving mechanisms including role-based access controls balance transparency objectives against legitimate commercial confidentiality requirements.

The data acquisition layer occupies the foundational position, serving as the primary interface between physical mining operations and digital information systems. Internet of Things sensors deployed across extraction sites, processing facilities, and transportation networks capture real-time operational parameters^32^. These devices generate continuous streams of production volumes, equipment status readings, environmental measurements, and location coordinates. Radio-frequency identification tags attached to material containers enable automated custody tracking without manual intervention. The critical challenge at this layer involves ensuring data authenticity at the point of origin—a concern we address through tamper-evident sensor designs and cryptographic attestation protocols.

Table 2 presents a systematic overview of functional responsibilities and enabling technologies across all architectural layers.

**Table 2:** Functional specifications of framework layers.

Layer	Primary function	Key technologies
Data acquisition	Multi-source information capture	IoT sensors, RFID, GPS tracking
Blockchain storage	Immutable record maintenance	Distributed ledger, smart contracts
Analysis processing	Data transformation and computation	Big data analytics, machine learning
Evaluation application	Performance assessment and visualization	Decision support systems, dashboards
Interface	Cross-layer communication	API gateways, middleware services

The blockchain storage layer receives validated data packets from acquisition components and commits them to the distributed ledger following consensus verification. Smart contracts automate business logic enforcement, triggering alerts when recorded parameters exceed predefined thresholds^33^. The analysis processing layer extracts actionable intelligence from accumulated historical records through statistical computation and pattern recognition algorithms. Finally, the evaluation application layer translates analytical outputs into comprehensible performance assessments accessible to diverse stakeholder groups.

Data flows upward through the architecture in a predominantly unidirectional manner, though feedback loops enable application-layer findings to inform acquisition configurations. The framework demonstrates strong extensibility characteristics—additional data sources integrate through standardized acquisition interfaces, while new evaluation modules plug into the application layer without architectural modification^34^. This adaptability proves essential given the heterogeneous nature of mining operations across different mineral types, geographic contexts, and regulatory jurisdictions. The modular design also accommodates evolving blockchain protocols, permitting consensus mechanism substitution as technology advances without wholesale system reconstruction.

### Data acquisition and on-chain storage mechanism

Effective traceability hinges upon reliable data capture at operational touchpoints throughout the mineral value chain. The acquisition strategy must accommodate diverse information sources while maintaining quality standards that justify subsequent blockchain immutability. Our framework implements a hybrid collection approach combining automated sensing with structured manual inputs where automation proves impractical or economically prohibitive^35^.

Automated data streams originate from three primary source categories. Weighbridge sensors and volumetric meters installed at extraction sites generate continuous production quantity measurements. Environmental monitoring stations capture emissions levels, water quality parameters, and noise readings essential for compliance verification. GPS-enabled tracking devices attached to transport vehicles and material containers provide real-time location updates and route documentation. Where conditions preclude sensor deployment—particularly during initial exploration phases or in remote artisanal mining contexts—trained personnel record observations through standardized mobile applications featuring validation constraints that minimize input errors^36^.

**Fig. 3:**
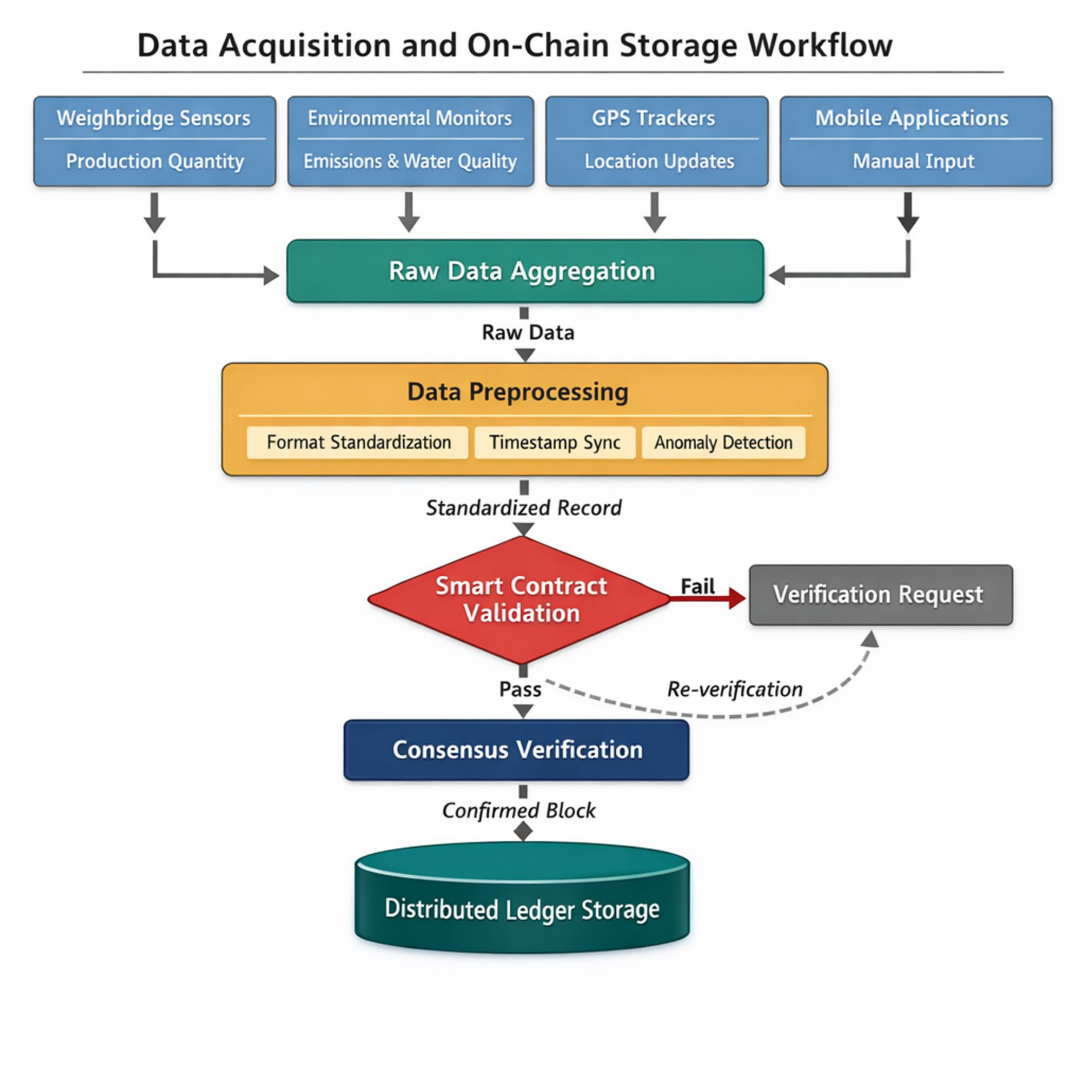
Data acquisition and on-chain storage workflow.

Raw data undergoes preprocessing before blockchain commitment. Figure 3 depicts the complete workflow from initial capture through final on-chain storage, highlighting intermediate transformation stages.

The preprocessing pipeline performs format standardization, timestamp synchronization, and anomaly detection. Outlier values exceeding statistical thresholds trigger verification requests rather than automatic rejection, recognizing that unusual readings sometimes reflect genuine operational conditions. The standardized data record *R* assumes a structured format:

8 $$R=\left\{ {ID,{T_{stamp}},{L_{geo}},{D_{payload}},{H_{prev}},{\sigma _{source}}} \right\}$$

where $ID$ represents unique transaction identifier, ${T_{stamp}}$ denotes synchronized timestamp, ${L_{geo}}$ indicates geographic coordinates, ${D_{payload}}$ contains operational data, ${H_{prev}}$ references preceding block hash, and ${\sigma _{source}}$ provides source authentication signature.

Smart contracts govern the on-chain commitment process through programmable validation rules^37^. Before accepting incoming data, contract logic verifies source authenticity, checks format compliance, and confirms temporal consistency with existing records. The validation function *V* returns boolean acceptance decisions based on multi-condition evaluation:

9 $$V\left( R \right)=\left\{ {\begin{array}{*{20}{c}} 1&{{\mathrm{if}}Auth\left( R \right) \wedge Format\left( R \right) \wedge Temporal\left( R \right){\mathrm{~}}0}&{{\mathrm{otherwise}}} \end{array}} \right.$$

Only records satisfying all validation criteria proceed to consensus processing and eventual ledger inclusion.

Privacy considerations demand careful attention given the commercially sensitive nature of production data. The framework implements role-based access controls that restrict information visibility according to stakeholder authorization levels. Regulatory authorities access comprehensive records, while commercial partners view only transaction-relevant subsets. Cryptographic techniques including zero-knowledge proofs enable verification of compliance claims without exposing underlying proprietary details^38^. This balanced approach reconciles transparency objectives with legitimate confidentiality requirements, encouraging participation from enterprises wary of competitive information exposure.

A critical limitation warrants explicit acknowledgment here. While blockchain technology ensures that data, once recorded on-chain, cannot be retroactively altered without detection, it cannot guarantee the authenticity of data at the point of origin. The framework implicitly assumes that information entering the system accurately reflects physical reality, yet several threats challenge this assumption. Sensor tampering may produce falsified readings that appear legitimate when committed to the blockchain. Human manipulation at data entry points can introduce fabricated information before any cryptographic protection takes effect. The physical-to-digital trust gap represents perhaps the most fundamental vulnerability: blockchain secures the digital record, but the correspondence between digital entries and physical events depends on mechanisms external to the distributed ledger itself.

We address these concerns through several complementary measures, though none provides absolute guarantees. Tamper-evident sensor designs incorporating hardware security modules make physical manipulation more difficult and detectable. Cross-validation protocols compare readings from independent sources, flagging discrepancies for investigation. Cryptographic attestation binds sensor identity to recorded data, creating audit trails for accountability. Nevertheless, sophisticated adversaries with physical access to data collection infrastructure may still circumvent these safeguards. Our evaluation framework therefore measures traceability performance under the assumption of generally honest behavior among system participants, and readers should interpret results accordingly. Future research might incorporate adversarial testing scenarios to assess system resilience under more challenging threat models.

### Traceability evaluation indicator system and assessment methods

The evaluation indicator system must capture the multifaceted nature of traceability performance across the complete mineral resource lifecycle. Drawing upon the theoretical foundations established in section “Theoretical framework for traceability evaluation”, we construct a hierarchical indicator architecture spanning three levels of analytical granularity^39^. The first-tier comprises five primary dimensions: traceability breadth, traceability depth, traceability precision, traceability timeliness, and data credibility. Each dimension decomposes into secondary indicators addressing specific performance aspects, which further subdivide into measurable tertiary metrics.

Table 3 presents the complete indicator system with corresponding measurement descriptions and indicator attributes.

**Table 3:** Traceability evaluation indicator system with justification.

First-tier indicator	Second-tier indicator	Third-tier indicator	Measurement description	Attribute	Relationship to traceability assurance
Traceability breadth (A1)	Lifecycle coverage (B1)	Phase completeness ratio (C1)	Documented phases / Total phases	Positive	Directly measures provenance reconstruction scope
	Stakeholder inclusion (B2)	Participant registration rate (C2)	Registered entities / Total entities	Positive	Indicates chain-of-custody completeness
Traceability depth (A2)	Information granularity (B3)	Data field density (C3)	Recorded fields / Required fields	Positive	Reflects richness of provenance documentation
	Historical retrievability (B4)	Query response success rate (C4)	Successful queries / Total queries	Positive	Measures accessibility of historical records (IT performance indicator)
Traceability precision (A3)	Location accuracy (B5)	Geographic deviation index (C5)	Average positioning error	Negative	Assesses spatial reliability of provenance claims
	Quantity accuracy (B6)	Measurement variance ratio (C6)	Variance from verified values	Negative	Evaluates volumetric reliability of recorded data
Traceability timeliness (A4)	Update frequency (B7)	Average refresh interval (C7)	Mean time between updates	Negative	Reflects currency of provenance information
	Query latency (B8)	Response time index (C8)	Average retrieval duration	Negative	System performance metric supporting operational efficiency
Data credibility (A5)	Source authenticity (B9)	Verification pass rate (C9)	Verified records / Total records	Positive	Measures proportion of records meeting authenticity criteria
	Consistency level (B10)	Cross-validation match rate (C10)	Consistent pairs / Total pairs	Positive	Assesses internal coherence of recorded information
	Tamper resistance (B11)	Blockchain confirmation ratio (C11)	On-chain records / Total records	Positive	Indicates proportion of records with blockchain integrity protection

Several indicators in this system measure IT system performance or data management quality rather than traceability assurance directly. Query response success rate (C4) and response time index (C8) primarily reflect technical infrastructure capabilities. Blockchain confirmation ratio (C11) indicates the extent of blockchain coverage but does not itself guarantee that confirmed records accurately represent physical reality. We retain these indicators because system performance constitutes a necessary precondition for effective traceability operations, even though it remains insufficient on its own.

The framework cannot measure certain aspects that matter for comprehensive traceability assurance. Pre-blockchain data authenticity, meaning the correspondence between physical events and initial digital records, lies outside the measurement scope. Adversarial resilience under coordinated attack scenarios receives no direct assessment. Long-term data preservation beyond the observation period remains unevaluated. Users should recognize these boundaries when interpreting evaluation results.

Indicator weights determination follows the Analytic Hierarchy Process methodology^40^. Pairwise comparison matrices capture expert judgments regarding relative importance at each hierarchical level. For a comparison matrix *A* with elements ${a_{ij}}$ representing the relative importance of indicator *i* over indicator *j*, the weight vector *W* derives from the principal eigenvector:

10 $$AW={\lambda _{max}}W$$

where ${\lambda _{max}}$ denotes the maximum eigenvalue. Consistency verification ensures judgment rationality through the consistency ratio:

11 $$CR=\frac{{CI}}{{RI}}=\frac{{\left( {{\lambda _{max}} - n} \right)/\left( {n - 1} \right)}}{{RI}}$$

Matrices achieving $CR<0.1$ satisfy acceptable consistency thresholds.

The fuzzy comprehensive evaluation method addresses inherent ambiguity in traceability assessment^41^. Performance ratings resist precise quantification, making fuzzy set theory particularly appropriate. We define the evaluation grade set as V = {v1, v2, v3, v4, v5} corresponding to five levels: Excellent (score range 85–100), Good (70–85), Moderate (55–70), Poor (40–55), and Very Poor (0–40).

#### Membership function specification

We employ trapezoidal membership functions to map indicator measurements to grade memberships. For positive indicators (where higher values indicate better performance), the membership degree to grade v_m_ follows:

For Excellent (v1): µ1(x) = 0, if x ≤ 70; (x − 70)/15, if 70 < x < 85; 1, if x ≥ 85.

For Good (v2): µ2(x) = 0, if x ≤ 55 or x ≥ 100; (x − 55)/15, if 55 < x ≤ 70; 1, if 70 < x < 85; (100 - x)/15, if 85 ≤ x < 100.

Similar trapezoidal functions apply to Moderate, Poor, and Very Poor grades with corresponding threshold shifts.

#### Negative indicator handling

For negative indicators (where lower values indicate better performance), we first apply inverse transformation: x’ = x_max_ - x + x_min_, where x_max_ and x_min_ represent the observed range bounds. The transformed value x’ then enters standard membership calculations as a positive indicator.

#### Worked numerical example

Consider the Source Authenticity indicator (C9) with observed verification pass rate of 91.2%. This positive indicator proceeds through membership calculation as follows:

Step 1: Apply membership functions.

- µExcellent(91.2) = 1 (since 91.2 ≥ 85).
- µGood(91.2) = (100 − 91.2)/15 = 0.587.
- µModerate(91.2) = 0.
- µPoor(91.2) = 0.
- µVery Poor(91.2) = 0.

Step 2: Normalize membership vector Raw vector: (1, 0.587, 0, 0, 0) Normalized: (0.630, 0.370, 0, 0, 0)

Step 3: Calculate indicator score Score = 0.630 × 95 + 0.370 × 80 + 0 × 65 + 0 × 50 + 0 × 30 = 59.85 + 29.60 = 89.45.

This calculation demonstrates how the 91.2% verification pass rate translates to a score of 89.45, placing it firmly in the Excellent category while acknowledging partial membership in the Good category due to distance from the 100% ideal.

#### Grade boundary sensitivity

The final comprehensive score of 81.2 lies 3.8 points above the Good/Moderate boundary (70) and 3.8 points below the Excellent/Good boundary (85). We examined whether small methodological changes could alter the grade classification. Shifting all grade boundaries downward by 5 points would elevate the rating to Excellent, while shifting boundaries upward by 5 points would maintain the Good classification. Substituting triangular membership functions for trapezoidal functions produced a comprehensive score of 80.7, preserving the Good grade. These tests suggest that the Good classification is reasonably stable but not immune to methodological variation.

The secondary-level fuzzy evaluation vector ${B_i}$ computes as:

12 $${B_i}={W_i} \circ {R_i}$$

where $\circ$ represents the fuzzy composition operator. Proceeding upward through the hierarchy, first-tier evaluation vectors emerge from secondary compositions:

13 $$B=W \circ R$$

The final comprehensive evaluation score *S* translates fuzzy outputs into numerical ratings using grade score vector $G={(95,80,65,50,30)^T}$:

14 $$S=B \times {G^T}$$

Grade classification follows predetermined thresholds: scores exceeding 85 indicate Excellent traceability, 70–85 suggests Good performance, 55–70 reflects Moderate capability, 40–55 denotes Poor status, and values below 40 signal Very Poor conditions^42^. This grading scheme provides actionable diagnostic information enabling targeted improvement interventions. The defuzzification process thus transforms abstract membership distributions into concrete performance categories readily interpretable by practitioners and regulators alike.

## Empirical analysis and validation

### Case selection and data sources

Empirical verification demands examination of real-world implementation contexts where theoretical constructs confront operational complexities. We selected Huaxin Mining Group Corporation, a large-scale integrated mining enterprise operating in northwestern China, as our case study subject. The selection rationale rests on several considerations: the enterprise manages multiple mineral commodities including copper, zinc, and associated precious metals; its operations span the complete value chain from exploration through refined product sales; and management expressed willingness to participate in blockchain pilot initiatives^43^. Annual extraction capacity exceeds 8 million metric tons of ore, processed through three beneficiation facilities before distribution to domestic smelters and international trading partners.

Prior to blockchain implementation, Huaxin relied on conventional enterprise resource planning systems supplemented by paper-based documentation at custody transfer points. This arrangement suffered from information fragmentation, reconciliation difficulties, and verification delays that complicated regulatory compliance demonstrations. The company recognized these limitations and collaborated with our research team to deploy a consortium blockchain network connecting operational nodes across extraction sites, processing plants, logistics centers, and sales offices.

System deployment proceeded through phased implementation spanning eighteen months. Initial infrastructure installation occurred during months one through six, focusing on node configuration and smart contract development. The subsequent integration phase connected existing sensor networks and enterprise databases to blockchain interfaces. Full operational status commenced in month thirteen, with the remaining period dedicated to system stabilization and personnel training^44^. Data collection for this study covers the twelve-month period following operational commencement, capturing seasonal variations and cyclical patterns inherent to mining activities.

**Fig. 4:**
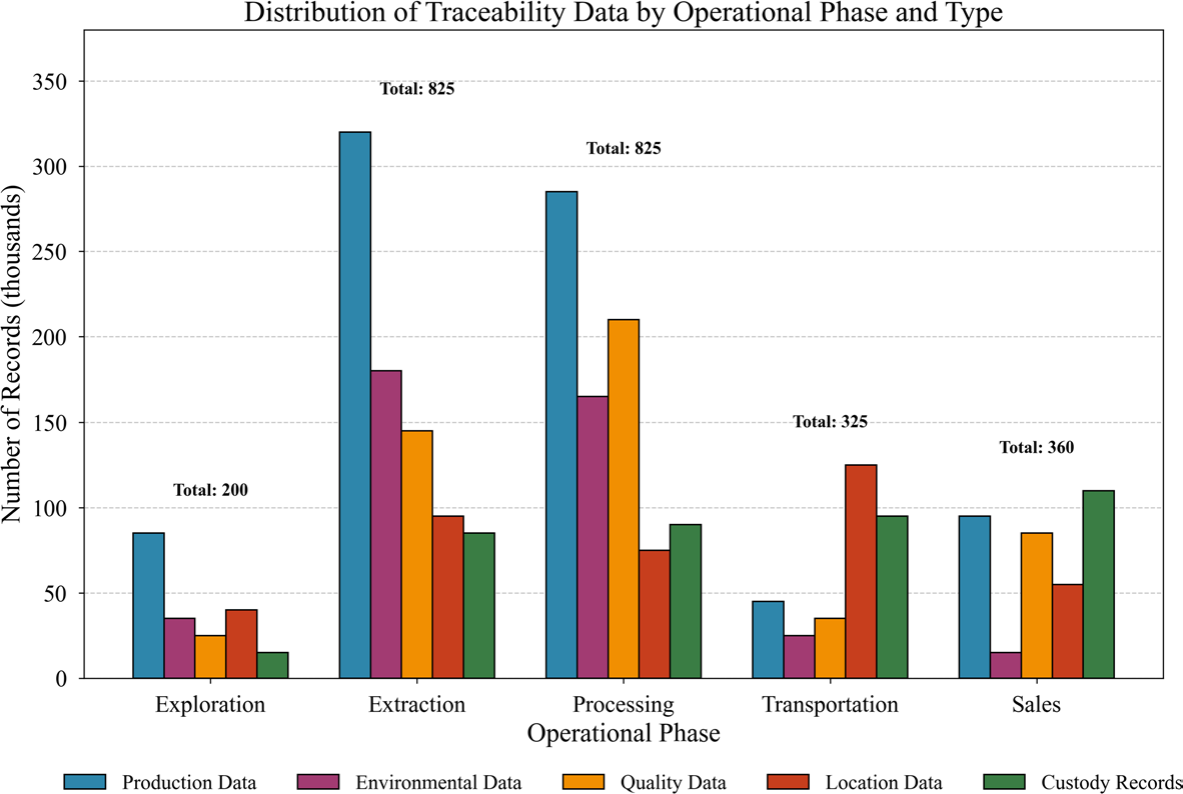
Distribution of traceability data by operational phase and type.

The dataset encompasses multiple information categories reflecting lifecycle comprehensiveness. Figure 4 illustrates the distribution of traceability records across operational phases and data types.

As Fig. 4 demonstrates, extraction and processing phases generate the highest record volumes, reflecting intensive monitoring requirements at these stages. Transportation records, though numerically fewer, contain richer attribute sets capturing location trajectories and handling conditions.

Table 4 presents descriptive statistics characterizing the sample dataset across key parameters.

**Table 4:** Descriptive statistics of sample data.

Variable	*N*	Mean	Std. Dev.	Min	Max
Daily transaction records	365	2,847	412.6	1,523	4,128
Block generation interval (s)	365	4.2	0.8	2.1	7.3
Data verification rate (%)	365	98.7	1.2	94.3	100.0
Query response time (ms)	365	127.4	23.5	89.0	215.0
Stakeholder participation rate (%)	12	87.3	5.4	78.2	94.6
Cross-validation match rate (%)	12	96.2	2.1	92.4	99.1
System uptime ratio (%)	12	99.4	0.3	98.7	99.9

**Fig. 5:**
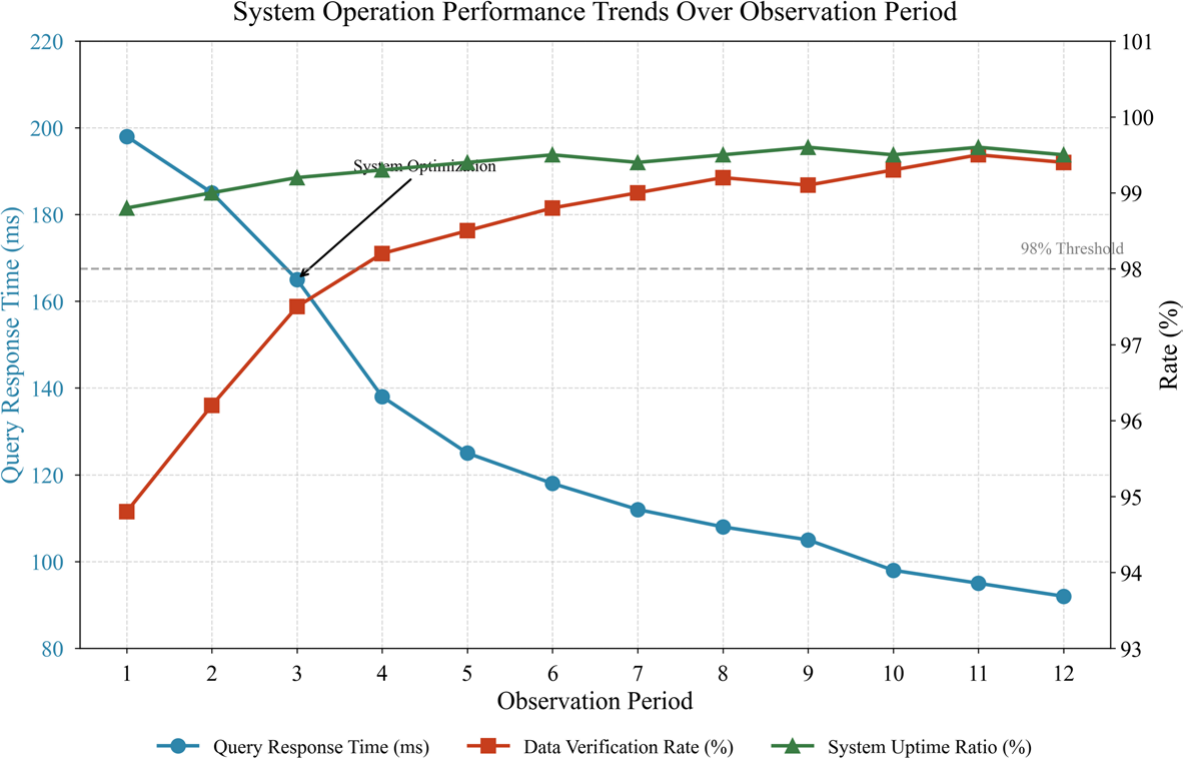
System operation performance trends over observation period.

The statistics reveal generally favorable operational patterns. Mean daily transaction volume of 2,847 records indicates substantial system activity, while low standard deviation in verification rates suggests consistent data quality maintenance^45^. Figure 5 depicts temporal trends in system performance metrics throughout the observation period.

Figure 5 shows progressive improvement in key performance indicators following initial deployment fluctuations. Query response times decreased notably after the third month as system optimization efforts took effect, while verification rates stabilized above 98% threshold. These preliminary findings suggest successful technical implementation, providing foundation for subsequent comprehensive traceability evaluation applying the indicator system developed in section “Traceability evaluation indicator system and assessment methods”.

#### Data aggregation and processing logic

The indicators in our evaluation framework draw upon data streams with heterogeneous time scales and sample sizes, necessitating clear aggregation procedures. Daily transaction records (*N* = 365 observations) provided inputs for timeliness and system performance indicators. Monthly aggregates (*N* = 12 observations) informed stakeholder participation and cross-validation metrics where underlying processes operate on longer cycles. Annual lifecycle completeness assessments drew upon cumulative records across the full observation period.

Mapping from raw operational logs to indicator scores proceeded through standardized transformation procedures. For ratio-based indicators (e.g., phase completeness ratio, verification pass rate), we calculated simple proportions from documented counts. For continuous measurements (e.g., geographic deviation, query response time), we computed arithmetic means with associated standard deviations to characterize central tendency and dispersion. Temporal indicators used median values rather than means to reduce sensitivity to outlier events such as system maintenance periods.

Normalization converted raw measurements to comparable scales before fuzzy membership calculation. Min-max normalization scaled values to [0, 100] ranges using observed extremes from the sample, acknowledging that this approach ties scores to the specific dataset rather than absolute performance standards. Alternative normalization using industry benchmark values (where available from published sources) produced similar rankings though slightly different absolute scores.

#### Generalization and scalability considerations

The single-case design limits direct generalization of quantitative findings to other contexts. The comprehensive score of 81.2 and blockchain contribution ratio of 47.3% reflect conditions specific to Huaxin Mining Group, including its commodity portfolio, geographic setting, organizational capabilities, and regulatory environment. Enterprises with different characteristics may achieve substantially different outcomes from blockchain implementation.

Several framework features support adaptation to diverse contexts. The modular indicator structure permits selective emphasis on dimensions most relevant to particular mineral types or regulatory requirements. Precious metal operations might weight data credibility more heavily given heightened provenance verification demands, while bulk commodity producers might prioritize timeliness given rapid inventory turnover. The hierarchical architecture accommodates indicator substitution without disrupting overall evaluation logic.

Scalability to larger or more complex supply chains remains untested within our empirical scope. Huaxin’s consortium blockchain connects approximately 40 organizational nodes; performance characteristics may differ substantially for networks spanning hundreds of participants or crossing multiple national jurisdictions. Transaction throughput limitations of current blockchain protocols could become binding constraints at larger scales, though layer-2 solutions and alternative consensus mechanisms offer potential remediation pathways.

Future validation across multiple enterprises, mineral types, and geographic regions would strengthen confidence in framework robustness. Comparative studies examining implementation in different regulatory environments would illuminate how institutional context moderates blockchain’s traceability contribution.

### Traceability evaluation results analysis

Applying the evaluation framework developed in section “Traceability evaluation indicator system and assessment methods”, we conducted comprehensive traceability assessment for Huaxin Mining Group based on the collected operational data. The evaluation process commenced with tertiary indicator measurement, followed by hierarchical aggregation through fuzzy comprehensive evaluation procedures. Expert panels comprising twelve industry specialists and academic researchers provided pairwise comparison judgments for weight determination via the Analytic Hierarchy Process.

The weight determination followed Analytic Hierarchy Process methodology with careful attention to procedural transparency and reproducibility. Expert panels comprising twelve specialists participated in pairwise comparison exercises. The panel composition included seven industry practitioners (mining engineers, supply chain managers, and quality assurance specialists with minimum eight years of relevant experience) and five academic researchers (holding doctoral degrees in mining engineering, information systems, or supply chain management with published research on traceability topics). Experts provided judgments independently without mutual consultation, and we aggregated individual comparison matrices using the geometric mean method to preserve reciprocal properties^40^ (Table 5).

**Table 5:** Pairwise comparison matrix for first-tier indicators.

	A1	A2	A3	A4	A5
A1 (Breadth)	1	4/3	7/6	3/2	1
A2 (Depth)	3/4	1	5/6	7/6	4/5
A3 (Precision)	6/7	6/5	1	4/3	1
A4 (Timeliness)	2/3	6/7	3/4	1	5/7
A5 (Credibility)	1	5/4	1	7/5	1

The consistency verification proceeded through standard calculations. Maximum eigenvalue λmax = 5.037 was obtained via power iteration. Consistency index CI = (λmax - n)/(*n* − 1) = (5.037–5)/(5 − 1) = 0.009. Random index RI = 1.12 for *n* = 5. Consistency ratio CR = CI/RI = 0.009/1.12 = 0.008 < 0.1, confirming acceptable judgment consistency.

The resulting first-tier weights are: Traceability Breadth (0.23), Traceability Depth (0.18), Traceability Precision (0.21), Traceability Timeliness (0.16), and Data Credibility (0.22). Complete pairwise comparison matrices for secondary and tertiary indicators, along with their consistency verification calculations, appear in Supplementary Material.

#### Sensitivity analysis

**Fig. 6:**
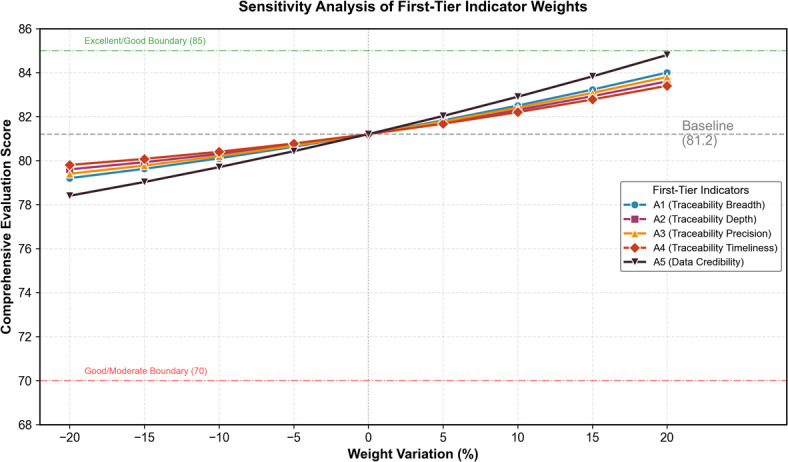
Sensitivity analysis of first-tier indicator weights.

To assess weight robustness, we conducted sensitivity analysis by systematically varying each first-tier weight within ± 20% of its baseline value while proportionally adjusting remaining weights. Figure 6 presents the sensitivity analysis results.

The analysis reveals that Data Credibility (A5) exerts the strongest influence on final scores, with a ± 20% weight change producing approximately ± 3.2 points variation in the comprehensive score. Traceability Timeliness (A4) shows the least sensitivity, with equivalent weight changes yielding only ± 1.8 points variation. Importantly, no weight perturbation within the tested range altered the final grade classification from “Good” to an adjacent category, suggesting reasonable stability of overall conclusions. Nevertheless, if Data Credibility weight were reduced by more than 35% (holding other ratios constant), the grade boundary would shift, indicating that assessment outcomes depend meaningfully on the credibility dimension’s assigned importance.

The composite weight for any tertiary indicator ${C_k}$ under secondary indicator ${B_j}$ and first-tier indicator ${A_i}$ computes as:

15 $$W_{{{C_k}}}^{{composite}}={W_{{A_i}}} \times {W_{{B_j}|{A_i}}} \times {W_{{C_k}|{B_j}}}$$

Fuzzy membership functions transformed raw performance measurements into grade distributions. For positive indicators, the membership degree to grade ${v_m}$ follows:

16 $${\mu _m}=\left\{ {\begin{array}{*{20}{c}} 1&{x \geqslant {s_m}{\mathrm{~}}\frac{{x - {s_{m+1}}}}{{{s_m} - {s_{m+1}}}}}&{{s_{m+1}}<x<{s_m}{\mathrm{~}}0}&{x \leqslant {s_{m+1}}} \end{array}} \right.$$

where ${s_m}$ represents the threshold value for grade *m*^46^. Table 6 summarizes the evaluation results across all first-tier and second-tier indicators.

**Table 6:** Summary of traceability evaluation results.

First-tier Indicator	Weight	Score	Second-tier Indicator	Weight	Score	Grade
Traceability breadth (A1)	0.23	82.6	Lifecycle coverage (B1)	0.55	85.3	Good
			Stakeholder inclusion (B2)	0.45	79.4	Good
Traceability depth (A2)	0.18	76.8	Information granularity (B3)	0.48	74.2	Good
			Historical retrievability (B4)	0.52	79.1	Good
Traceability precision (A3)	0.21	71.4	Location accuracy (B5)	0.46	68.5	Moderate
			Quantity accuracy (B6)	0.54	73.8	Good
Traceability timeliness (A4)	0.16	84.7	Update frequency (B7)	0.42	82.3	Good
			Query latency (B8)	0.58	86.5	Excellent
Data credibility (A5)	0.22	88.3	Source authenticity (B9)	0.38	91.2	Excellent
			Consistency level (B10)	0.35	86.7	Excellent
			Tamper resistance (B11)	0.27	85.4	Good

**Fig. 7:**
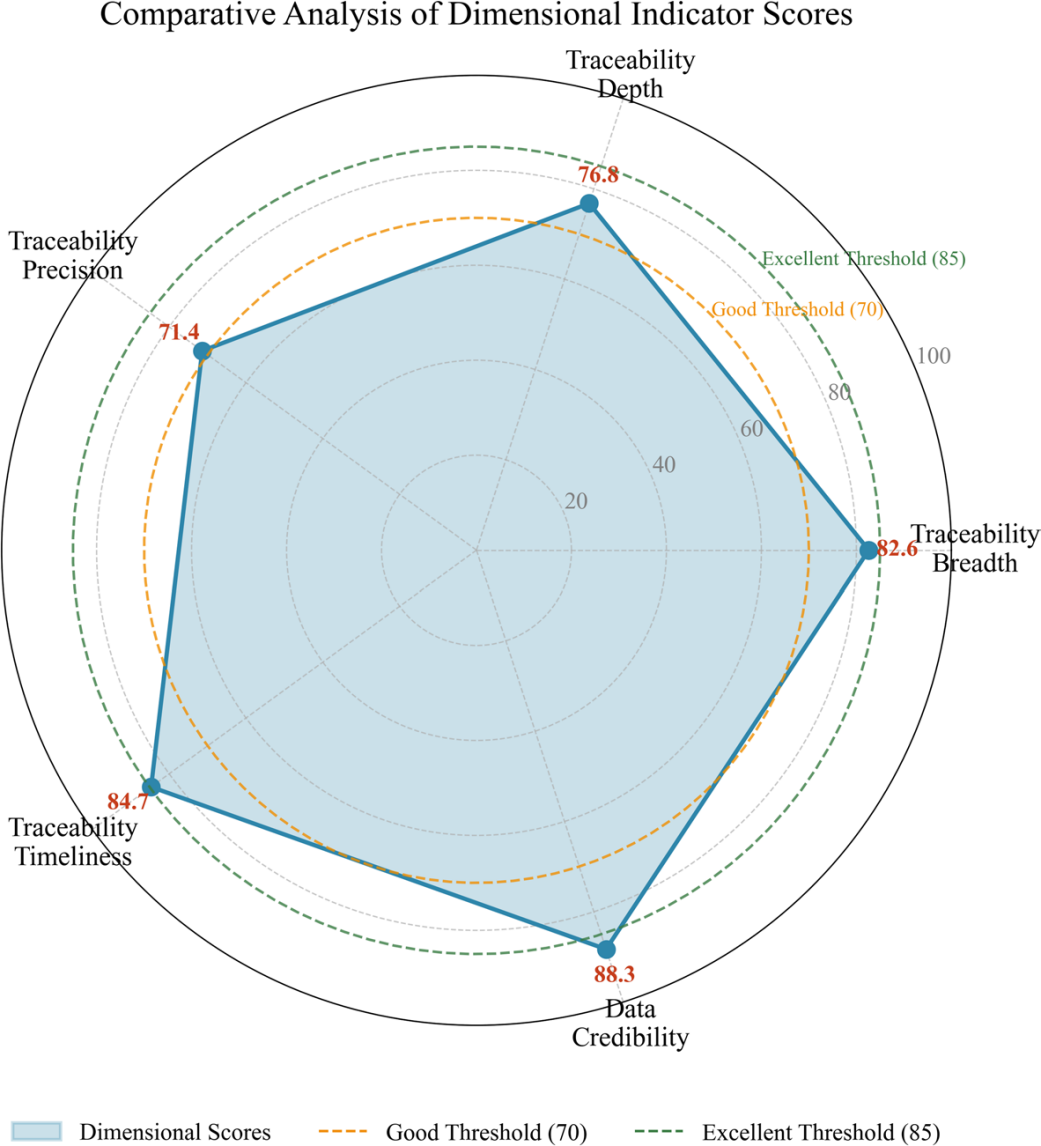
Comparative analysis of dimensional indicator scores.

Figure 7 presents a radar chart visualization comparing dimensional performance scores, revealing notable variation across traceability dimensions.

As Fig. 7 demonstrates, Data Credibility achieves the highest dimensional score (88.3), reflecting blockchain’s inherent strengths in ensuring information integrity^47^. Traceability Timeliness similarly performs well, benefiting from automated data capture and rapid consensus confirmation. Conversely, Traceability Precision registers the lowest score (71.4), indicating room for improvement in measurement accuracy—particularly regarding geographic positioning during transportation phases.

The comprehensive evaluation score derives from weighted aggregation of dimensional scores:

17 $${S_{comprehensive}}=\mathop \sum \limits_{{i=1}}^{5} {W_{{A_i}}} \times {S_{{A_i}}}=0.23\left( {82.6} \right)+0.18\left( {76.8} \right)+0.21\left( {71.4} \right)+0.16\left( {84.7} \right)+0.22\left( {88.3} \right)$$

**Fig. 8:**
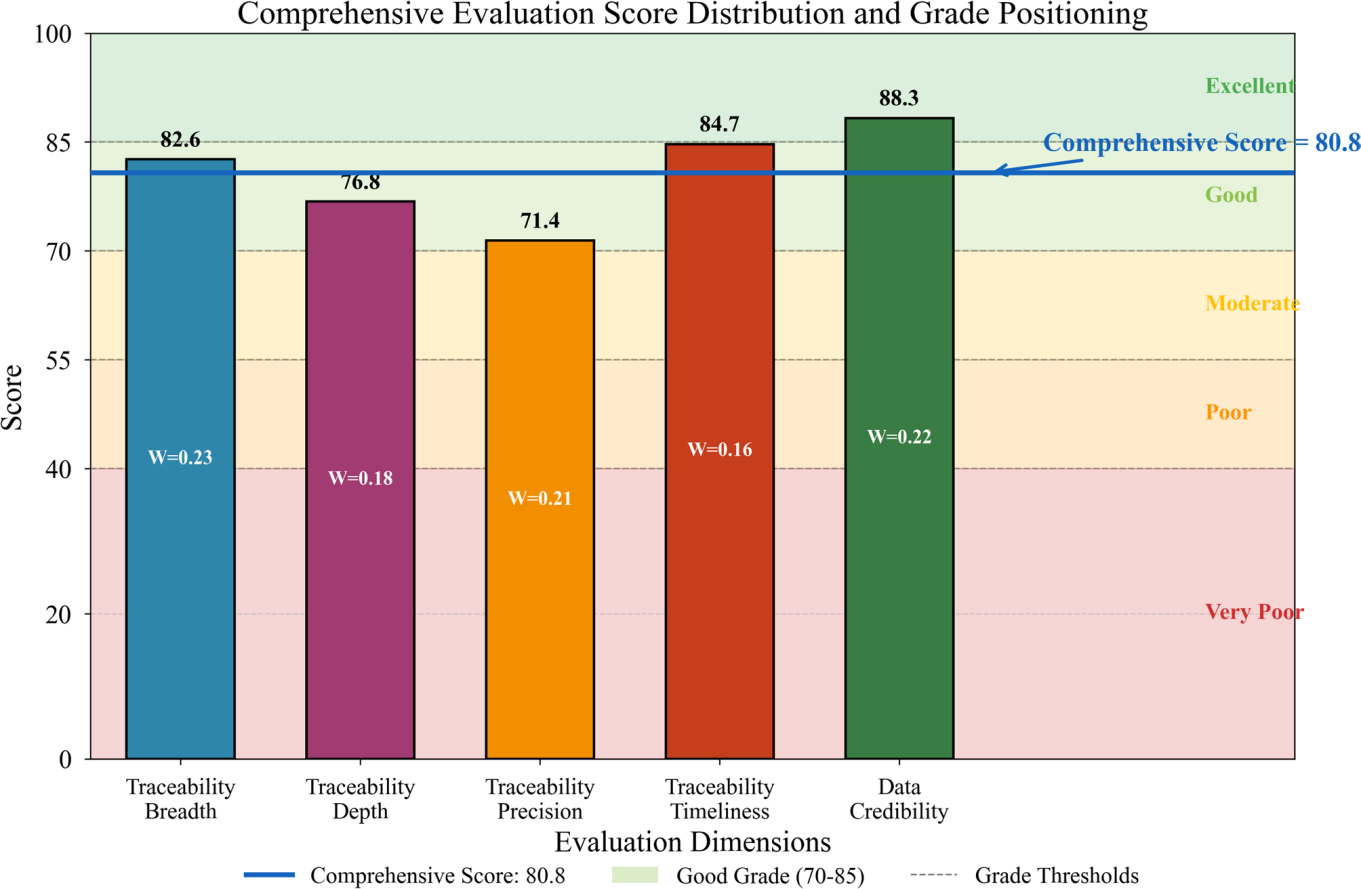
Comprehensive evaluation score distribution and grade positioning.

Computing this expression yields ${S_{comprehensive}}=81.2$, placing Huaxin’s traceability performance within the “Good” grade category. Figure 8 illustrates the score distribution and grade positioning.

The evaluation reveals several weak links warranting management attention. Location accuracy deficiencies stem from GPS signal attenuation in underground mining environments and mountainous terrain along transportation routes. Stakeholder inclusion gaps arise from incomplete onboarding of smaller subcontractors and third-party logistics providers who lack technical capacity for blockchain integration^48^. Information granularity limitations reflect legacy system constraints that restrict data field richness at certain processing stages.

Targeted improvement recommendations address these identified weaknesses. First, deploying hybrid positioning systems combining GPS with inertial navigation units would enhance location tracking reliability in challenging environments. The expected precision improvement follows:

18 $$\Delta P={P_{hybrid}} - {P_{GPS}}=\alpha \cdot{P_{INS}}+\left( {1 - \alpha } \right)\cdot{P_{GPS}} - {P_{GPS}}$$

where $\alpha$ represents the fusion coefficient optimized through Kalman filtering. Second, establishing simplified mobile interfaces and providing technical assistance programs would facilitate broader stakeholder participation. Third, upgrading legacy database schemas and implementing middleware translation layers would enable richer information capture without wholesale system replacement. These interventions, prioritized according to cost-benefit considerations, offer pathways toward advancing from “Good” to “Excellent” traceability performance.

### Comparative analysis with traditional traceability models

Rigorous evaluation of blockchain-enabled traceability demands systematic comparison against conventional approaches. We identified Dongfang Mining Corporation, an enterprise of comparable scale and operational scope to Huaxin, as the control group. Dongfang continues operating traditional traceability infrastructure comprising centralized database systems, paper-based custody documentation, and periodic manual audits. Both enterprises process similar mineral commodities (copper, zinc, and associated precious metals) and serve overlapping market segments, rendering comparison meaningful. Data collection from the control group covered the identical twelve-month observation period, ensuring temporal consistency.

#### Comparability assumptions and limitations

The comparative design rests on several assumptions that warrant explicit acknowledgment. Beyond comparable scale and commodity mix, structural and contextual factors may influence traceability performance independently of system design. Huaxin operates primarily in plateau terrain with established road networks, while Dongfang’s operations include mountainous regions with more complex transportation routes. If observed differences in location accuracy or trace-back time partly reflect logistical complexity rather than traceability system effectiveness, our estimates of blockchain contribution would be biased upward.

Regulatory enforcement intensity may also differ between the two enterprises’ operating jurisdictions, potentially affecting documentation practices and verification thoroughness through channels unrelated to technological infrastructure. Additionally, workforce experience with digital systems varies: Huaxin’s personnel received targeted blockchain training during implementation, whereas Dongfang’s staff maintain established routines with traditional documentation. We cannot fully disentangle technology effects from human capital and organizational learning effects within our research design.

#### Statistical assumption verification

The independent samples t-test assumes approximately normal distribution of compared variables or sufficient sample sizes for robustness to non-normality. We assessed distributional properties through Shapiro-Wilk tests applied to both groups’ trace-back time and accuracy measurements. Trace-back time data exhibited moderate right skewness (Shapiro-Wilk *p* = 0.031 for Huaxin, *p* = 0.047 for Dongfang), suggesting departure from strict normality.

Given sample sizes exceeding 300 observations for each variable, the Central Limit Theorem supports t-test robustness despite non-normality. Nevertheless, we conducted confirmatory non-parametric analysis using the Mann-Whitney U test. Results confirmed statistical significance of between-group differences (U = 2,847, *p* < 0.001 for trace-back efficiency; U = 3,102, *p* < 0.001 for data accuracy), corroborating t-test conclusions.

Readers should interpret comparative findings with awareness that unobserved enterprise-level differences may influence statistical precision and that causal attribution to blockchain technology specifically requires assumptions about comparability that our observational design cannot fully verify.

**Fig. 9:**
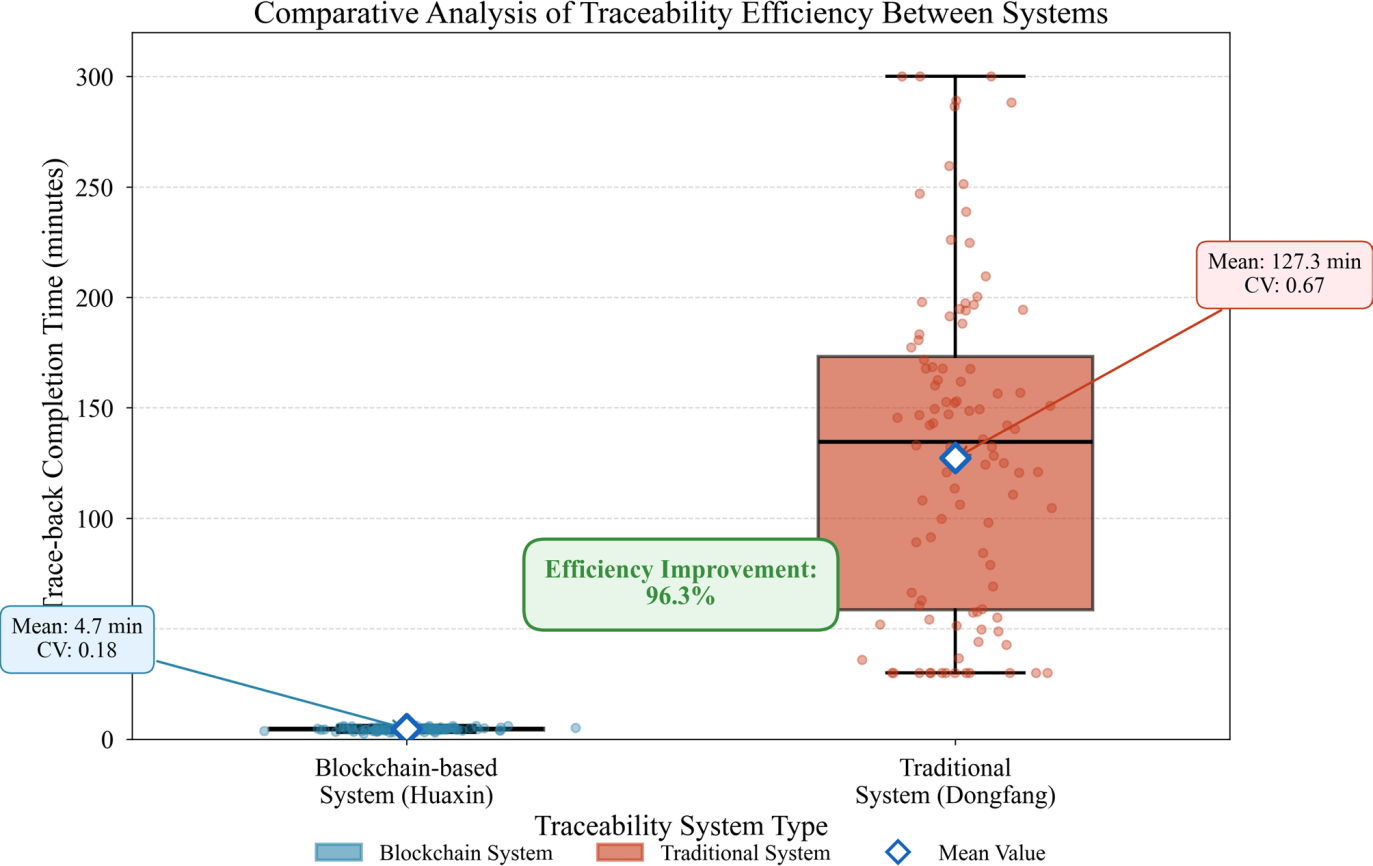
Comparative analysis of traceability efficiency between systems.

Traceability efficiency represents a critical performance dimension reflecting system responsiveness to inquiry demands. We measured complete trace-back duration—the time required to reconstruct full provenance from finished product to original extraction point. Huaxin’s blockchain system achieved mean trace-back completion in 4.7 min, contrasting sharply with Dongfang’s average of 127.3 min under traditional procedures. Figure 9 depicts the efficiency distribution comparison across randomly sampled traceability requests.

As Fig. 9 illustrates, blockchain-based tracing demonstrates not only superior mean performance but also substantially reduced variance. The coefficient of variation for blockchain tracing stands at 0.18 versus 0.67 for traditional methods, indicating more predictable and reliable response characteristics. This consistency proves particularly valuable during regulatory inspections when time-sensitive verification demands arise unexpectedly.

**Fig. 10:**
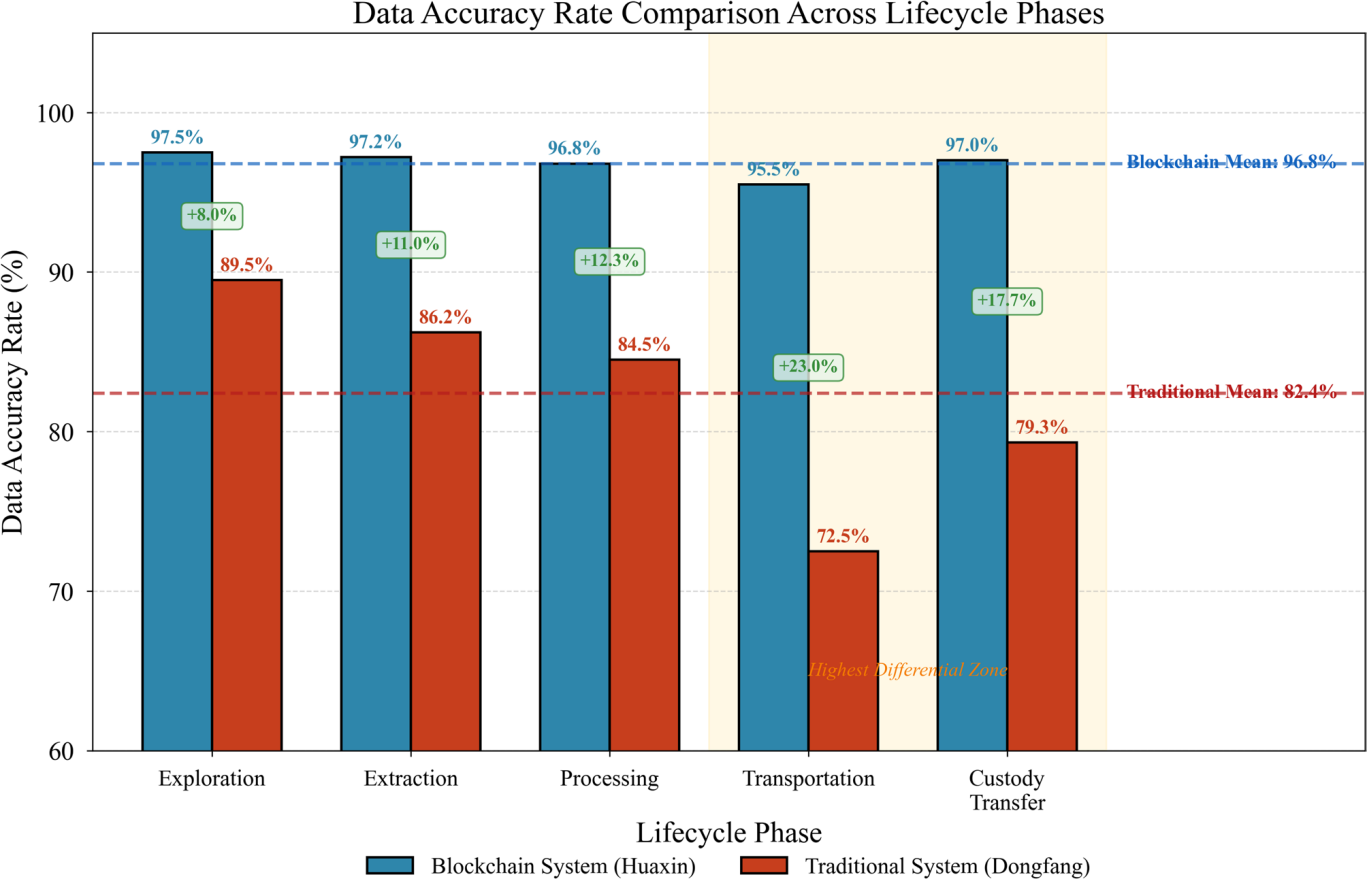
Data accuracy rate comparison across lifecycle phases.

Data accuracy constitutes another fundamental comparison dimension. We conducted cross-validation exercises matching recorded information against physical audit findings for 500 randomly selected material batches from each enterprise. The blockchain system yielded 96.8% concordance between digital records and physical reality, whereas traditional documentation achieved only 82.4% accuracy^49^. Figure 10 presents the accuracy rate comparison across different lifecycle phases.

Figure 10 reveals that accuracy differentials widen particularly during transportation and custody transfer phases—precisely where traditional paper-based documentation faces greatest vulnerability to loss, damage, and deliberate falsification. Blockchain immutability and automated capture mechanisms address these weaknesses directly.

Statistical hypothesis testing formally validates observed performance differences. We employed the independent samples t-test to assess whether mean differences achieve statistical significance. The test statistic computes as:

19 $$t=\frac{{{{\bar {X}}_{blockchain}} - {{\bar {X}}_{traditional}}}}{{\sqrt[{}]{{\frac{{s_{1}^{2}}}{{{n_1}}}+\frac{{s_{2}^{2}}}{{{n_2}}}}}}}$$

For traceability efficiency, the calculated t-value of 23.47 far exceeds the critical threshold at $\alpha =0.01$ significance level, confirming that blockchain superiority reflects genuine systematic differences rather than sampling fluctuation. Similarly, accuracy rate comparisons yield t = 8.92, again statistically significant.

Cost analysis reveals more nuanced patterns. Blockchain implementation entailed substantial initial investment—approximately 12.8 million yuan for infrastructure, development, and training. Traditional system maintenance costs approximately 2.3 million yuan annually. However, the blockchain system reduces ongoing operational expenses through automation and error reduction. The net present value calculation over a ten-year horizon follows:

20 $$NPV= - {I_0}+\mathop \sum \limits_{{t=1}}^{{10}} \frac{{{{({C_{traditional}} - {C_{blockchain}})}_t}+{B_{indirect}}}}{{{{(1+r)}^t}}}$$

where ${I_0}$ represents initial investment, *C* denotes annual costs, ${B_{indirect}}$ captures indirect benefits from reduced disputes and regulatory penalties, and *r* indicates discount rate^50^. Computing with estimated parameters yields positive NPV of 8.7 million yuan, indicating favorable long-term economics despite high upfront expenditure.

The contribution degree of blockchain technology to traceability enhancement can be quantified through decomposition analysis:

21 $$\eta =\frac{{{S_{blockchain}} - {S_{traditional}}}}{{{S_{maximum}} - {S_{traditional}}}} \times 100\%$$

Substituting evaluation scores from section “Traceability evaluation results analysis” and applying equivalent assessment to Dongfang yields $\eta =47.3\%$, suggesting that blockchain technology accounts for nearly half of the potential improvement achievable from baseline traditional performance toward theoretical maximum.

Beyond quantifiable metrics, blockchain implementation generates broader societal benefits. Enhanced traceability strengthens regulatory enforcement capacity, deterring illegal extraction and tax evasion behaviors that undermine public revenue. Environmental compliance verification becomes more credible, supporting sustainable resource governance objectives. Consumer and downstream manufacturer confidence in material provenance increases, potentially commanding price premiums for verified responsible sourcing. These diffuse benefits, though challenging to monetize precisely, reinforce the case for blockchain adoption across the mineral resource sector.

## Discussion

The integration of blockchain technology with mineral resource traceability evaluation rests on a fundamental alignment between technological characteristics and governance demands. Mineral value chains suffer from information asymmetries that erode trust among stakeholders—extractors, processors, regulators, and end consumers occupy distinct positions with divergent incentives regarding data disclosure. Blockchain architecture directly confronts this challenge through distributed consensus mechanisms that eliminate reliance on any single trusted intermediary. The cryptographic linking of records creates accountability structures where falsification becomes computationally prohibitive and detectable. This technological-governance fit explains why blockchain holds particular promise for mineral traceability contexts where traditional centralized approaches have persistently struggled.

Our evaluation framework advances beyond existing research contributions in several respects. Previous studies predominantly examined blockchain implementation feasibility without developing systematic assessment methodologies capable of quantifying traceability performance improvements. The hierarchical indicator system proposed herein addresses this gap by operationalizing abstract traceability concepts into measurable metrics spanning breadth, depth, precision, timeliness, and credibility dimensions. The fuzzy comprehensive evaluation approach accommodates inherent measurement uncertainty that rigid scoring schemes ignore. Perhaps more significantly, the framework maintains adaptability across different mineral types and operational contexts through modular indicator selection—an enterprise extracting precious metals faces distinct traceability priorities compared to bulk commodity producers, and our architecture accommodates such variation.

The empirical findings carry implications extending beyond the immediate case context. The substantial performance differential between blockchain-enabled and traditional traceability systems—particularly the 47.3% contribution ratio calculated in section “ Comparative analysis with traditional traceability models”—suggests that technological intervention meaningfully advances governance objectives rather than merely substituting one documentation method for another. Yet the evaluation also revealed persistent weaknesses in location accuracy and stakeholder inclusion, reminding us that technology alone cannot resolve all traceability challenges. Human and organizational factors retain decisive influence over system effectiveness.

Regarding regulatory policy considerations, our case-based findings may inform discussions about blockchain-based traceability requirements, though they do not constitute sufficient evidence for specific policy recommendations. The observed performance improvements in a single enterprise context suggest potential relevance for policy design in high-risk mineral commodity contexts where provenance verification concerns are acute. Conflict minerals, environmentally sensitive extraction zones, and cross-border trade settings represent domains where policymakers might consider examining blockchain traceability options.

Any policy deliberations should recognize limitations of our evidence base. Prescriptive regulatory mandates would require broader empirical support across multiple jurisdictions and enterprise types than our single-case study provides. Cost-benefit assessments at sector-wide scales remain necessary before concluding that blockchain traceability mandates would generate net social benefits. Smaller operators lacking technical capacity might face disproportionate compliance burdens under uniform requirements, suggesting that graduated implementation timelines and shared infrastructure arrangements merit consideration in policy design.

We present these observations as potential inputs to policy discourse rather than as definitive recommendations. Regulatory authorities possess contextual knowledge about their specific jurisdictional circumstances that our research cannot substitute.

Enterprises contemplating traceability system investments should recognize the front-loaded cost structure our analysis revealed. Initial expenditure is substantial, but operational savings and indirect benefits accumulate over time, eventually yielding favorable returns. Strategic patience proves essential—abandoning blockchain initiatives before benefits materialize would forfeit realized investments without capturing long-term value.

Industry standardization efforts should prioritize interoperability specifications enabling cross-enterprise data exchange. Currently, proprietary blockchain implementations risk creating fragmented ecosystems where traceability chains break at organizational boundaries. Common data schemas, consensus protocol compatibility, and mutual recognition arrangements would multiply network effects and accelerate sectoral adoption.

Several obstacles constrain broader blockchain deployment in mineral resource contexts. Technical challenges include scalability limitations when transaction volumes surge during peak production periods, energy consumption concerns associated with certain consensus mechanisms, and integration difficulties with legacy enterprise systems. Institutional barriers encompass unclear legal status of blockchain records as evidentiary documents, jurisdictional conflicts in cross-border mining operations, and resistance from stakeholders benefiting from current opacity. Cost considerations deter resource-constrained enterprises, particularly those operating in developing regions where mining activity concentrates but digital infrastructure remains underdeveloped. Addressing these multifaceted challenges demands coordinated action across technological, regulatory, and capacity-building domains.

## Conclusion

This investigation addressed the pressing need for rigorous traceability evaluation mechanisms in mineral resource governance by developing a comprehensive blockchain-enabled assessment framework. The research traversed theoretical foundation establishment, framework architecture design, indicator system construction, evaluation methodology formulation, and empirical validation through case study analysis. Each component builds upon preceding elements to form an integrated analytical apparatus capable of systematically measuring and enhancing traceability performance across mineral value chains.

Several conclusions emerge from this investigation, though each should be understood within the scope limitations we have acknowledged. First, the proposed four-layer framework architecture provides a coherent technical structure bridging operational data capture with performance assessment in our case context. While the modular design appears adaptable to diverse mineral types and organizational contexts, such adaptability awaits confirmation through multi-case validation before strong generalizability claims become warranted.

Second, the hierarchical indicator system spanning traceability breadth, depth, precision, timeliness, and data credibility dimensions operationalizes governance objectives into measurable metrics. Within the Huaxin case, these indicators discriminated meaningfully between performance levels and identified specific improvement targets. Whether the same indicator set proves equally diagnostic in different operational environments remains an empirical question for future research.

Third, the fuzzy comprehensive evaluation methodology demonstrated suitability for traceability assessment in this context, accommodating measurement ambiguity while producing interpretable results. The sensitivity analyses suggest reasonable robustness of grade classifications, though alternative methodological choices could yield moderately different scores.

Fourth, comparative analysis revealed substantial performance differences between blockchain-enabled and traditional traceability approaches in the studied enterprises. Statistical testing confirmed significance of observed differentials, yet readers should recall our earlier discussion of comparability assumptions and the possibility that unobserved factors contribute to between-group differences.

Regarding contributions, we offer case-based empirical evidence illustrating how blockchain technology can enhance traceability in a mineral extraction context. The framework provides a methodological template that others might adapt to their circumstances, pending validation of its applicability beyond our specific case. We refrain from claiming to have established general theoretical foundations or advanced research frontiers, as such claims would exceed what single-case evidence can support.

The practical implications remain correspondingly bounded. Regulatory authorities might find our evaluation approach informative as one input among many when considering traceability assessment tools, though sector-wide applicability requires broader testing. Mining enterprises facing similar contexts to Huaxin may find the diagnostic framework useful for identifying improvement priorities. Industry-wide standardization efforts would benefit from accumulated evidence across multiple implementations before adopting any particular evaluation scheme as a certification foundation.

Future progress toward transparent and accountable resource governance will require sustained research effort extending well beyond what this single study contributes. Our work represents one step in an ongoing journey rather than a destination.

Candid acknowledgment of research limitations remains essential. The single-case empirical design, while permitting detailed examination, constrains generalizability claims—different mineral commodities, geographic regions, and regulatory environments may yield divergent results. The twelve-month observation window captures operational patterns but cannot assess long-term system sustainability or performance trajectory beyond initial implementation phases. Certain potentially relevant indicators—particularly those addressing environmental and social dimensions of responsible sourcing—received insufficient attention in the current framework and warrant incorporation in refined versions.

Future research directions present abundant opportunities. Cross-chain interoperability mechanisms enabling seamless data exchange between distinct blockchain networks would address fragmentation concerns limiting current implementations. Artificial intelligence integration promises automated anomaly detection, predictive analytics, and adaptive evaluation parameter optimization that static rule-based systems cannot achieve. Dynamic evaluation approaches incorporating real-time performance monitoring rather than periodic assessment would enhance responsiveness to emerging issues. Expanding empirical validation across multiple enterprises, mineral types, and jurisdictions would strengthen confidence in framework robustness. These avenues collectively point toward increasingly sophisticated blockchain-enabled governance systems advancing mineral resource traceability toward its full potential.

## Data Availability

All data generated and analyzed during the current study are available from the corresponding author upon reasonable request.

## Abbreviations

IoT: Internet of things
RFID: Radio-frequency identification
GPS: Global positioning system
GIS: Geographic information system
API: Application programming interface
AHP: Analytic hierarchy process
SHA-256: Secure Hash Algorithm 256-bit
NPV: Net present value
CR: Consistency ratio
CI: Consistency index
RI: Random index
ERP: Enterprise resource planning
